# Release of functional peptides from mother's milk and fortifier proteins in the premature infant stomach

**DOI:** 10.1371/journal.pone.0208204

**Published:** 2018-11-29

**Authors:** Søren D. Nielsen, Robert L. Beverly, Mark A. Underwood, David C. Dallas

**Affiliations:** 1 Nutrition Program, School of Biological and Population Health Sciences, College of Public Health and Human Sciences, Oregon State University, Corvallis, OR, United States of America; 2 Department of Pediatrics, University of California, Davis, Sacramento, CA, United States of America; National Cancer Institute at Frederick, UNITED STATES

## Abstract

Digestion of milk proteins in the premature infant stomach releases functional peptides; however, which peptides are present has not been reported. Premature infants are often fed a combination of human milk and bovine milk fortifiers, but the variety of functional peptides released from both human and bovine milk proteins remains uncharacterized. This study applied peptidomics to investigate the peptides released in gastric digestion of mother’s milk proteins and supplemental bovine milk proteins in premature infants. Peptides were assessed for homology against a database of known functional peptides—Milk Bioactive Peptide Database. The peptidomic data were analyzed to interpret which proteases most likely released them from the parent protein. We identified 5,264 unique peptides from bovine and human milk proteins within human milk, fortifier or infant gastric samples. Plasmin was predicted to be the most active protease in milk, while pepsin or cathepsin D were predicted to be most active in the stomach. Alignment of the peptide distribution showed a different digestion pattern between human and bovine proteins. The number of peptides with high homology to known functional peptides (antimicrobial, angiotensin-converting enzyme-inhibitory, antioxidant, immunomodulatory, etc.) increased from milk to the premature infant stomach and was greater from bovine milk proteins than human milk proteins. The differential release of bioactive peptides from human and bovine milk proteins may impact overall health outcomes in premature infants.

## Introduction

Human milk composition is evolutionarily optimized to provide essential nourishment for the term infant [1]. Human milk proteins provide a balanced source of amino acids that are essential for the infant’s rapid growth. However, milk proteins provide more than the ideal amino acids for infants. In vitro studies suggest that peptides encrypted within parent milk proteins possess a variety of bioactive functions, including antimicrobial [2], angiotensin-converting enzyme (ACE) inhibition [3], immunomodulation [4, 5], antioxidant [6], opioid [7] and calcium delivery [8]. Many of these bioactive peptides are released from milk proteins during digestion within the mammary gland by native milk proteases and by proteases within the infant gut. Our previous work demonstrated that mother’s milk contains a coordinated array of proteases and antiproteases that together release specific peptides from milk proteins within the mammary gland [9–13], and that in term infants, both milk proteases and infant digestive proteases release functional peptides within the stomach [14–16]. These encrypted peptides may be functional units with biological effects within the infant that evolved to be released based on the specificity of proteases in the normal term mother’s milk and the term infant’s gut.

During early years of the evolutionary timescale, infants born prematurely (< 37 weeks gestational age) were unlikely to have exerted much selective pressure on milk composition and structure, as they rarely survived. Premature infants have a lower protein digestion capacity compared with term infants due to their lower gastric pepsin and intestinal protease activity [17, 18]. Therefore, the peptides released from milk proteins during premature infant digestion may be different from those released in term infants, which could impact the functional contribution of the peptides that affect infant health and development.

Premature infants are typically not provided a single source of protein. Human milk is preferred over bovine milk-based formulas due to its positive health outcome associations, including reduced risk of necrotizing enterocolitis (NEC) [19, 20] and sepsis [21], improved cognitive skills [22] and decreased time to hospital discharge [23]. However, the energy and protein content of human milk alone do not ensure optimal growth in preterm infants. Therefore, human milk fed to preterm infants is typically fortified to meet their protein needs, which range from 2.5 to 4 g protein/kg body weight/day depending on gestational age at birth and day of life [24]. Human milk fortifiers (HMFs) can derive from either human or bovine milk [25, 26]. While limited evidence suggests that human milk-based fortifiers may reduce risk of NEC [27], bovine fortifiers are commonly used due to their lower cost and higher availability [28]. The different amino acid sequences of bovine proteins may lead to differential degradation in the infant. The processing—particularly the heat treatments used to ensure sterility—of milk proteins to prepare fortifiers can change protein structure, which can alter the susceptibility of the protein to proteolysis [29, 30] and, hence, release of bioactive peptides.

A few studies that investigated the digestion of human milk proteins and bovine fortifier proteins using in vitro and rhesus macaque models found similar rates of digestion based on gel electrophoresis protein profiling [31, 32], yet whether the specific peptides released differ between these protein sources has not been determined, particularly in human infants.

To determine which functional peptides premature infants are exposed to during digestion of consumed milk, we used peptidomics to analyze the peptides released from human and bovine milk proteins based on homology with known functional peptides in our recently created Milk Bioactive Peptide Database [33]. We assessed the peptidomics data to determine which proteases were most likely responsible for their release. This initial work can lead to insights on how the food type can affect the bioactive potential of peptides within protein fragments that affect the preterm infant’s overall health.

## Materials and methods

### Participants and sample collection

Human milk samples and their matching gastric milk samples were collected from five mother–premature infant pairs, as approved by the UC Davis Institutional Review Board. Written consents were obtained from all mothers. Milks from mothers of infants in the Neonatal Intensive Care Unit (NICU) of the UC Davis Medical Center were collected by pumping on-site or at home with sterile breast pumps and stored immediately at –20°C. Milks pumped at home were transported to the NICU on ice and stored at –40°C. Milks were transported from the NICU to the laboratory on dry ice and stored at –80°C. The infants of these same mothers were sampled for their stomach contents at 2 h after initiation of feeding the mother’s milk. Due to their need for more concentrated nourishment, these premature infants were fed mother’s milk enriched with a bovine milk protein-based fortifier (Abbott Similac HMF powder (1 0.9-g packet per 25 mL human milk). Samples were taken via suction from already in-place oro- or naso-gastric feeding tubes. Infants selected for this study had no known digestive functional problems. Infants ranged from 24 to 32 weeks gestational age and from 11 to 45 days of life. After collection, gastric samples were immediately stored at –40°C. Samples were transferred to the laboratory on dry ice and stored at –80°C.

### Sample preparation

Aliquots of milk and gastric aspirates (between 8 and 20 μL) from the five mother–infant pairs were thawed on ice for approximately 30 min. A sample of the HMF was dissolved in nanopure water at the same concentration as was added to the infant feeds. HMF was processed according to the steps described for the milk and gastric samples. To remove the milk fat, samples were centrifuged at 3,000 x *g* for 10 min at 4°C and placed on ice while the infranate were collected by pipette. Milk proteins were precipitated from the skimmed milk by addition of 240 g/L trichloroacetic acid (1:1 sample:solution). After mixing, the samples were centrifuged (4,000 x *g* for 10 min at 4°C) and the peptide-containing supernates were collected.

Trichloroacetic acid, salts, oligosaccharides and lactose were removed from the peptide solution and peptides were extracted via C18 reverse-phase preparative chromatography 96-well plates (Glygen, Columbia, MD) as previously described [34]. The purified peptide solutions were frozen at –80°C and lyophilized using a freeze dry system (Labconco FreeZone 4.5 L, Kansas City, MO). After drying, the samples were rehydrated in 0.1% formic acid in water to their original volume.

### Liquid chromatography nano-electrospray ionization mass spectrometry

The liquid chromatography separation was performed on a Waters nanoAcquity Ultra-Performance Liquid Chromatography (UPLC) (Waters Corporation, Milford, MA, USA) with a nanospray source. One microliter of each sample was loaded onto a 180 μm × 20 mm, 5-μm bead 2G nanoAcquity UPLC trap column (reverse phase) for online desalting and enrichment, and then onto a 100 μm × 100 mm, 1.7-μm bead Acquity UPLC Peptide BEH C18 column (Waters) for analytical separation. The LC eluent was initially set to 97% solvent A (0.1% FA in water) and 3% solvent B (99.9% ACN, 0.1% FA) with a flow rate of 500 nL/min. Then, the LC gradient increased from 3–10% solvent B over 3 min, then 10–30% solvent B over 99 min, then 30–90% solvent B over 3 min, then 90% solvent B for 4 min, then 90–3% solvent B over 1 min and finally kept at 3% solvent B for 10 min. Each sample run was followed by a 30-min column wash.

The peptides were profiled with a Thermo Scientific Orbitrap Fusion Lumos mass spectrometer. Spectra were obtained in positive-ionization mode with an electrospray voltage set to 2,400 V. The MS scan range was 400–1500 m/z at a resolution of 120K. The fragmentation mode was set to collision-induced dissociation and the collision energy was 35%. The MS cycle time was 3 s with data-dependent analysis and automated precursor peak selection. Precursor ions were excluded after one fragmentation for 60 s and exclusion within a 10 ppm mass error window. Precursors were selected for fragmentation based on the following criteria: most intense peaks, ion-intensity threshold 5.0 × 103 and charge state 2–7. Fragments were detected with the ion trap with an automatic scan range.

Spectra were analyzed by database searching in Thermo Proteome Discoverer (v2.1.0.81) using an in-house human and bovine milk protein sequence database as previously described in detail [35]. Potential modifications allowed included phosphorylation of serine and threonine and oxidation of methionine. Proteome Discoverer percolator was used to discriminate between correct and incorrect spectrum identifications using a decoy database search. Based on this, only peptides identified with high confidence (*P* < 0.01) were included in the results (FDR < 0.01). Peptide sequences with multiple modifications were grouped into a single peptide for counts and ion intensity. “Counts” represents the number of unique peptide sequences identified in a sample and “ion intensity” represents the area under the curve of the eluted peak. The peptide ion intensity, is used as an approximation of the amount of peptides in the samples. The data have been deposited to the jPOST repository [36] (ID: PXD010502).

### Data analysis

Peptides were mapped to the parent protein sequence of β-casein, α_s1_-casein and osteopontin using an in-house tool (PepEx) [37], available at http://mbpdb.nws.oregonstate.edu/pepex/. This mapping provides a visual overview of the position of peptides released within the parent protein sequence by totaling the ion intensity of each amino acid from the peptidomic data. The sequences of bovine and human milk proteins were aligned using the Protein BLAST alignment tool at blast.ncbi.nlm.nih.gov. This data was combined with the Pepex output to compare the digestion of proteins from either human or bovine origin. The protein hydrophobicity distribution was determined using ProtScale (https://web.expasy.org/protscale/) which employs the method described by Tanford [38]. The hydrophobicity was calculated as an average of seven amino acids and a linear weight variation with 25% relative weight of the amino acids at the window edge.

Excel was used to create tables of amino acids present in the P1 and P1’ positions at the N- and C-terminal cleavage sites of each peptide identified. The nomenclature for a protein cleavage site was formulated by Schecter and Berger [39]. P1 is the first amino acid positioned before a cleavage site. P1’ is the first amino acid positioned after a cleavage site.

Proteasix, an online tool that predicts enzymes involved in peptide cleavage using the cleavage site-specificity matrices for proteases within the Merops protease cleavage site database [40], was used to predict the proteases involved in peptide cleavage within the five milk and five gastric samples. The peptide data were searched against the following proteases: cathepsin D (CTSD; P07339), neutrophil elastase (ELANE; P08246), thrombin (F2; P00734), kallikrein 6 (KLK 6; Q92876), kallikrein 11 (KLK 11; Q9UBX7), plasmin (PLG; P00747) and pepsin (PGA3; P0DJD8). Proteasix searches the database of known peptide cleavages against the peptidomic data, identifying instances where the exact cleavage site has previously been observed (experimental data) and attributed to a specific enzyme. The results from Proteasix are given as experimentally known cleavages (observed) and predicted cleavages based on the cleavage site amino acids positioned at P4P3P2P1-P1’P2’P3’P4’. As predictions based on eight amino acids might be too stringent, Proteasix allows for up to 3 mismatches (from high to low confidence). However, in all cases cleavage site restrictions are obeyed. All predicted and observed cleavages were combined in the final results [39].

Identified peptides in milk, HMF and gastric samples were examined for homology with literature-identified bioactive peptides using the Milk Bioactive Peptide Database (MBPDB, http://mbpdb.nws.oregonstate.edu/) [33], which is a comprehensive collection of all milk bioactive peptides. The search was performed as a sequence search that searches for bioactive peptides matching the input peptide amino acid sequence. The similarity threshold was set to 80% and the amino acid scoring matrix was set to identity. “Get extra output” was selected to obtain the specific percentage similarity between the query sequence and the bioactive peptide sequence.

### Protein concentration

The protein concentration in human milk and gastric samples were measured with the bicinchoninic acid assay protein assay.

### Statistical analysis

Analyses were carried out using the statistical program RStudio version 1.0.136. A linear mixed model with Tukey's HSD *post hoc* test was used to adjust for multiple comparisons between bovine and human milk peptides. Significant difference was defined as *P* < 0.05. Results are presented as least square means ± standard error.

## Results

Demographic details for the five mother-infant pairs are presented in Table 1. Each 25 mL of human milk was fortified with 0.25 g of bovine milk proteins. Sampling of human milk for this analysis was performed prior to HMF addition for infant feeding.

**Table 1:** Demographic information for the mother–infant pairs sampled for milk and gastric contents.

	Gestational age, wk	Postnatal age, d	Birth weight, g	Birth length, cm	Mother’s age, y	Infant sex (F/M), no.
Mean ^a^	26 ± 2	28 ± 6	940 ± 180	34 ± 2	34 ± 2	3/2
Range of values	24–32	11–45	620–1610	28–39	30–40	

^a^ ± standard error

The protein concentration of human milk was 15.9 ± 1.0 mg/mL and the gastric aspirates two hours after feeding human milk fortified with bovine milk proteins (10 mg/mL) contained 17.3 ± 1.8 mg/mL.

### Peptide profile

Peptide profiling by mass spectrometry identified a total of 5,264 unique peptides deriving from human and bovine milk proteins (14,413 when counting modification variants) across all human milk, HMF, and gastric samples. From the total number of identified peptides, 1,722 and 3,399 originated from bovine and human milk proteins, respectively. One hundred and thirty-eight peptides derived from protein regions that have identical sequences for human and bovine milk proteins and, thus, their origin could not be distinguished. The peptides that were indistinguishable were mostly attributed to relatively low abundance milk proteins such as actin, xanthine dehydrogenase, fatty acid-binding protein, and lipoprotein lipase.

The five human milk samples contained an average of 719.6 ± 77.2 peptides with a total ion intensity 7.62 × 10^11^ ± 1.19× 10^11^. Bovine milk-based HMF measured alone (not mixed with human milk) contained 538 peptides with a total ion intensity of 1.1 × 10^11^, whereas infant gastric samples two hours after initiation of feeding contained an average of 1,720.8 ± 108.2 peptides accounting for a total ion intensity of 1.67 × 10^12^ ± 2.85 × 10^11^. Of the average number of peptides identified in the gastric sample, 980.8 ± 101.0 derived from human milk proteins with 9.21 × 10^11^ ± 1.49 × 10^11^ total ion intensity and 691.6 ± 7.2 peptides derived from bovine milk proteins with 7.39 × 10^11^ ± 1.35 × 10^11^ total ion intensity. The number of peptides identified from human milk proteins increased significantly from human milk before feeding to the gastric two hours after initiation of feeding.

**Fig 1:**
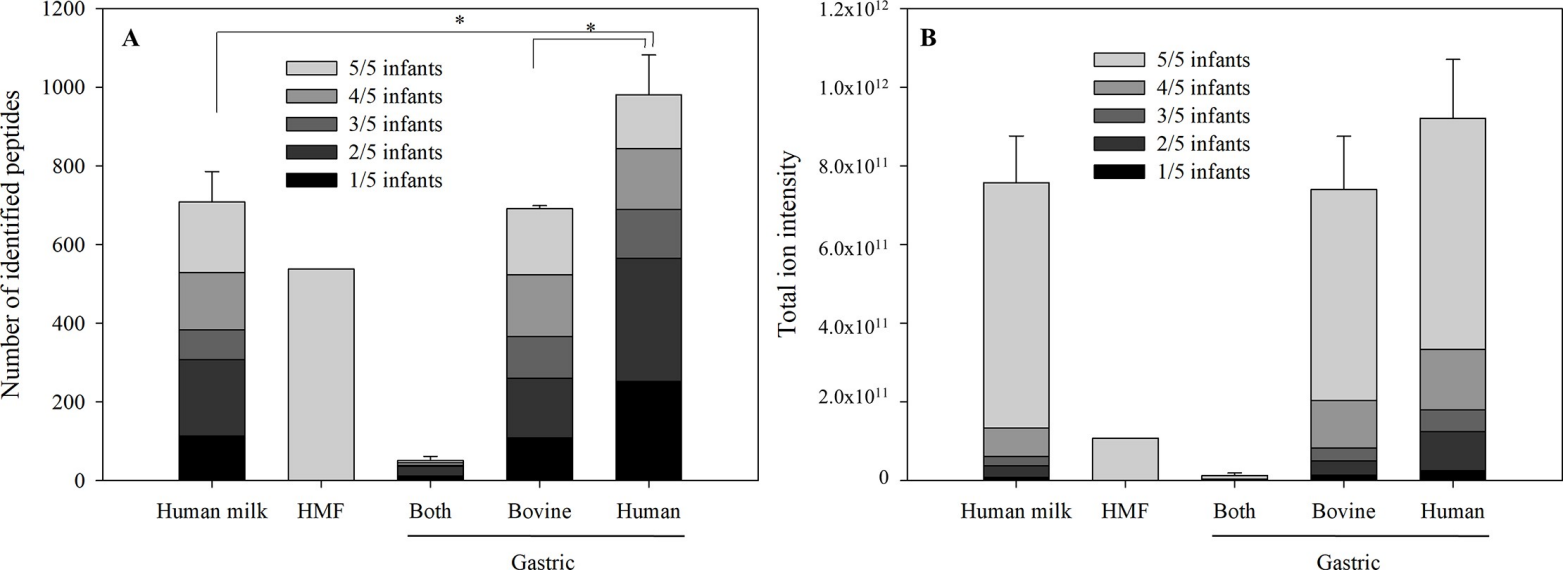
Total number of peptides identified in human milk and infant gastric. Total count (A) and abundance (B) of peptides identified in unfortified human milk and preterm infant gastric digests of fortified human milk from either bovine milk proteins, human milk proteins or either of the two. The graph is divided into sections of different shades of grey (from black to light grey) for the number of infants the peptides were identified in. Black represents peptides only identified in one infant and the lightest shade of grey representing peptide found in all 5 infants. Results are shown as mean ± standard error.

One hundred eighty-five peptides were found in all five milk samples, making up 25.7% of the total number of peptides identified in human milk (Fig 1A). Three hundred ten peptides were found in all five infant gastric samples: 168 from bovine milk, 137 from human milk, and 5 that were indistinguishable (Fig 1A). Combined, these peptides accounted for 18% of the total number of peptides identified in the gastric samples. A greater number of peptide sequences were thus released from human milk proteins (as represented by the lower percentage of conserved sequences) than from bovine fortifier proteins from the standardized HMF following preterm gastric digestion.

The majority of the peptide content (by ion intensity) in both milk and gastric samples was accounted for by peptide sequences present in samples from all five of the infants (82.4% and 67.7% respectively) (Fig 1B). In the gastric samples, 63.8% of the human milk protein-derived peptide ion intensity and 72.5% of the bovine milk protein-derived peptide ion intensity was comprised of peptide sequences present in all samples. For both human and bovine proteins, most of the abundance derived from peptides present in all samples, but human milk peptides were slightly less conserved across infants.

Several of the human milk peptides identified in the human milk samples were also identified in the gastric samples from the corresponding infant. Peptides identified in both milk and gastric accounted for 21.7 ± 5.2% of the total number of peptides deriving from human milk proteins identified in the gastric, and accounted for 32.2 ± 6.1% of the peptide total ion intensity. The list of peptides can be found in S1 Table.

**Fig 2:**
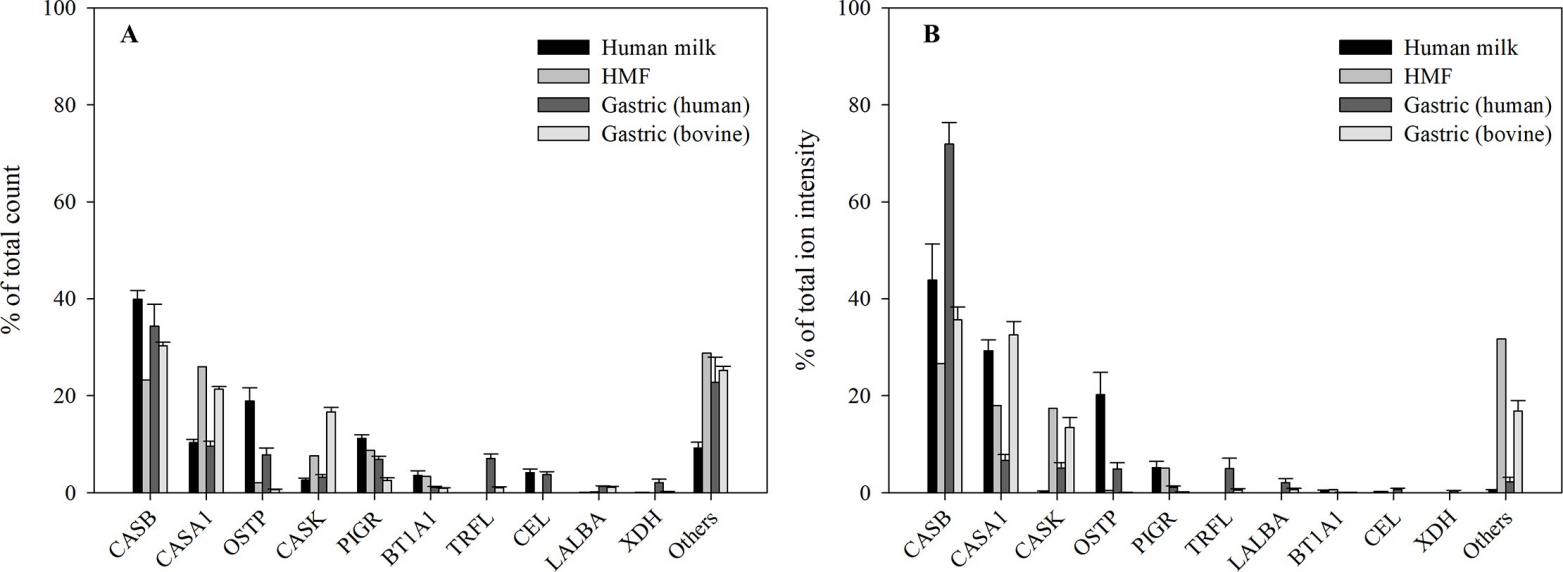
Relative number of peptides identified from proteins in human milk and infant gastric. Relative count (A) and relative ion intensity (B) of peptides identified in human milk and preterm infants gastric from either bovine milk proteins (bovine), human milk proteins (human), sorted according to the total peptide release among all samples. Results are shown as mean ± standard error. CASB, β-casein; OSTP, osteopontin; CASA1, αs1-casein; PIGR, polymeric immunoglobulin receptor; CEL, bile salt-activated lipase; BT1A1, butyrophilin subfamily 1 member A1; TRFL, lactoferrin; CASK, κ-casein; XDH, xanthine dehydrogenase/oxidase; LALBA, α-lactalbumin.

Next, we examined the extent of digestion of individual proteins. To compare between sample and feed types, we represented each protein’s peptides relative to the total human or bovine peptide profile in that sample (Fig 2). In HMF, peptides deriving from β-casein contributed with the highest ion intensity (26.7% of total), whereas the highest number of peptides was identified from α_s1_-casein (26.2% of total). In both the milk and gastric samples, most peptides where identified from β-casein. Human β-casein made up 39.9% of the total number of identified peptides and 43.8% of the total ion intensity of peptides in the human milk samples, and 33% of total number of peptides and 71.2% of total ion intensity of human milk protein-derived peptides in the gastric samples. Bovine β-casein made up 30.3% of the total number of peptides and 35.6% of the total ion intensity of bovine milk protein-derived peptides in the gastric samples. For human milk protein-derived peptides in the gastric samples, the next two highest contributors were α_s1_-casein and osteopontin, and each accounted for less than one-third of β-casein’s count and less than one-tenth of its ion intensity. The distribution of bovine protein contribution to the peptide counts and ion intensity was more equivalent for the top three proteins (β-casein, α_s1_-casein, and κ-casein) compared with human milk proteins. In addition to proteins present in both human and bovine milk, peptides from proteins unique to bovine milk were identified in the gastric samples, including β-lactoglobulin (count, ion intensity; 10.2%, 3.9%) and α_s2_-casein (7.7%, 3.3%).

### Peptide distribution

**Fig 3:**
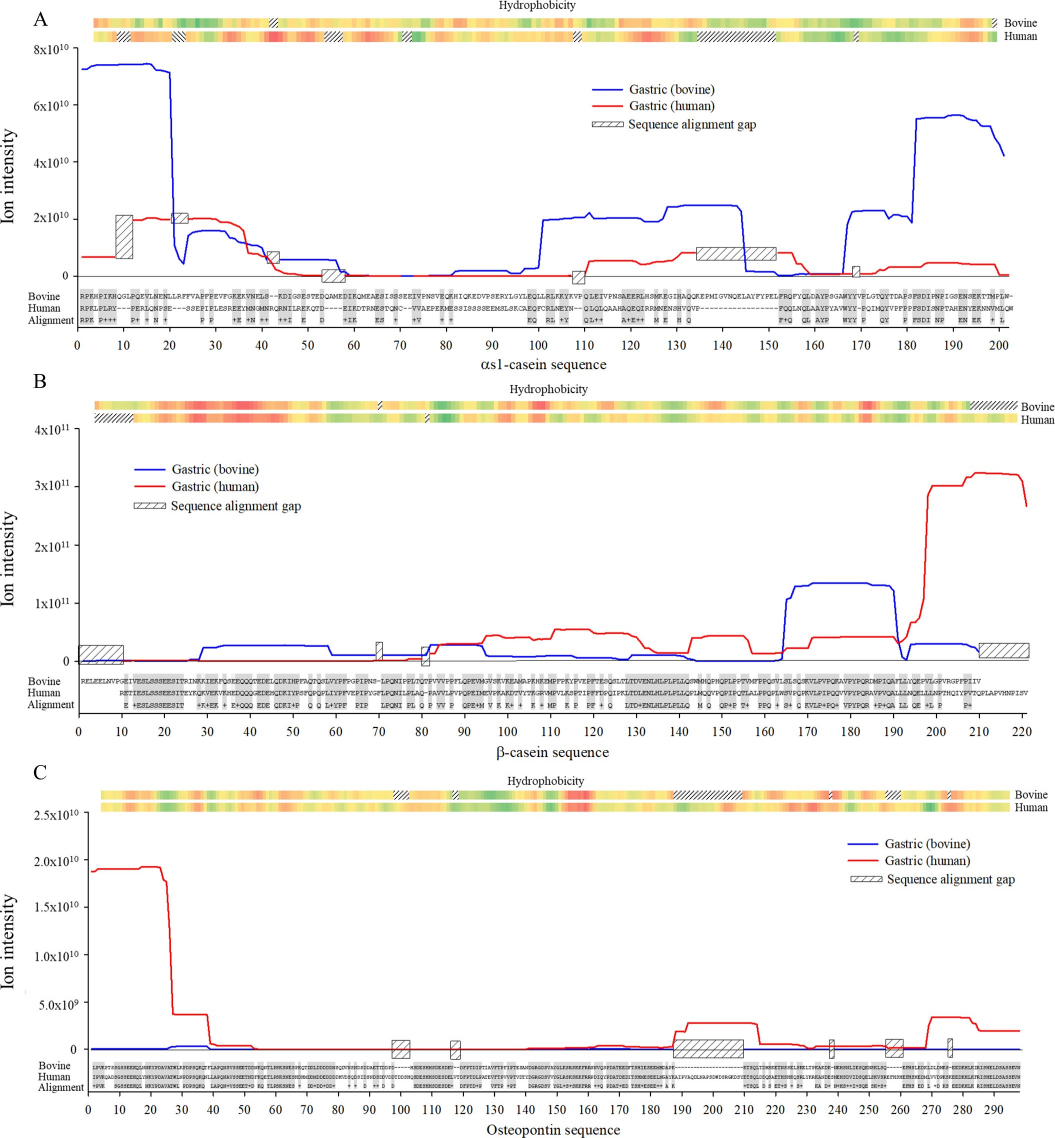
Mapping of identified peptide on milk proteins. Total ion intensity of peptides identified in infant gastric samples from bovine and human milk β-casein (A), α_s1_-casein (B) and osteopontin (C). The ion intensity of each peptide also present in the human milk samples or HMF were subtracted from the gastric values prior to mapping to the sequence of the proteins. Results are shown as means, *n* = 5. The hydrophobicity score is shown as a heat map with green as the most hydrophobic, red as the most hydrophilic.

The protein sequences of β-casein, α_s1_-casein and osteopontin were aligned between the human and bovine protein sequences using the online bioinformatics software Protein BLAST. The peptides identified in the gastric samples were then distributed across the aligned protein sequence according to their ion intensity using the PepEx software. Ion intensity of peptides also identified in human milk or HMF were subtracted from those present in the gastric samples to better represent only peptides released in the stomach. The hydrophobicity score was added above the peptide distribution as it impacts protein folding and potential accessibility for proteases (Fig 3). In general, the observed cleavage sites were mostly different between bovine and human milk proteins. The largest cleavage site in bovine α_s1_-casein by ion intensity was between Leu^20^(P1)—Leu^21^(P1’) (amino acid number is counted without the signal peptide). This site is not present in the human α_s1_-casein sequence. Similarly, the largest cleavage site in human α_s1_-casein (Asn^35^-Arg^36^) is not present in bovine α_s1_-casein. Several of the most abundant sequences of cleavage sites were conserved between the two species yet not cleaved to the same degree, e.g. the highly cleaved site Phe^179^-Ser^180^ in bovine α_s1_-casein is present as Phe^149^-Ser^150^ in the human protein, but it is not highly cleaved in the human protein despite their highly similar hydrophobicity patterns. Similar results were found for β-casein. The largest cleavage site of bovine β-casein was Leu^163^-Ser^164^, which was not present in the human β-casein sequence; and the largest site of human β-casein was Leu^187^-Leu^190^, which was present in the bovine β-casein sequence. Interestingly, the major cleavage sites of osteopontin were mostly conserved between the two species as was their hydrophobicity pattern (note: despite the sequence similarities, none of the osteopontin peptide sequences were identical for bovine and human. The largest cleavage site in bovine osteopontin was Phe^38^-Leu^39^, which was present in human osteopontin as Leu^38^-Leu^39^; whereas the largest in human osteopontin was Thr^26^-Trp^27^, which was exactly conserved in bovine osteopontin. An additional site was conserved at Ala^25^-Thr^26^ for both species.

### Enzyme cleavage

**Fig 4:**
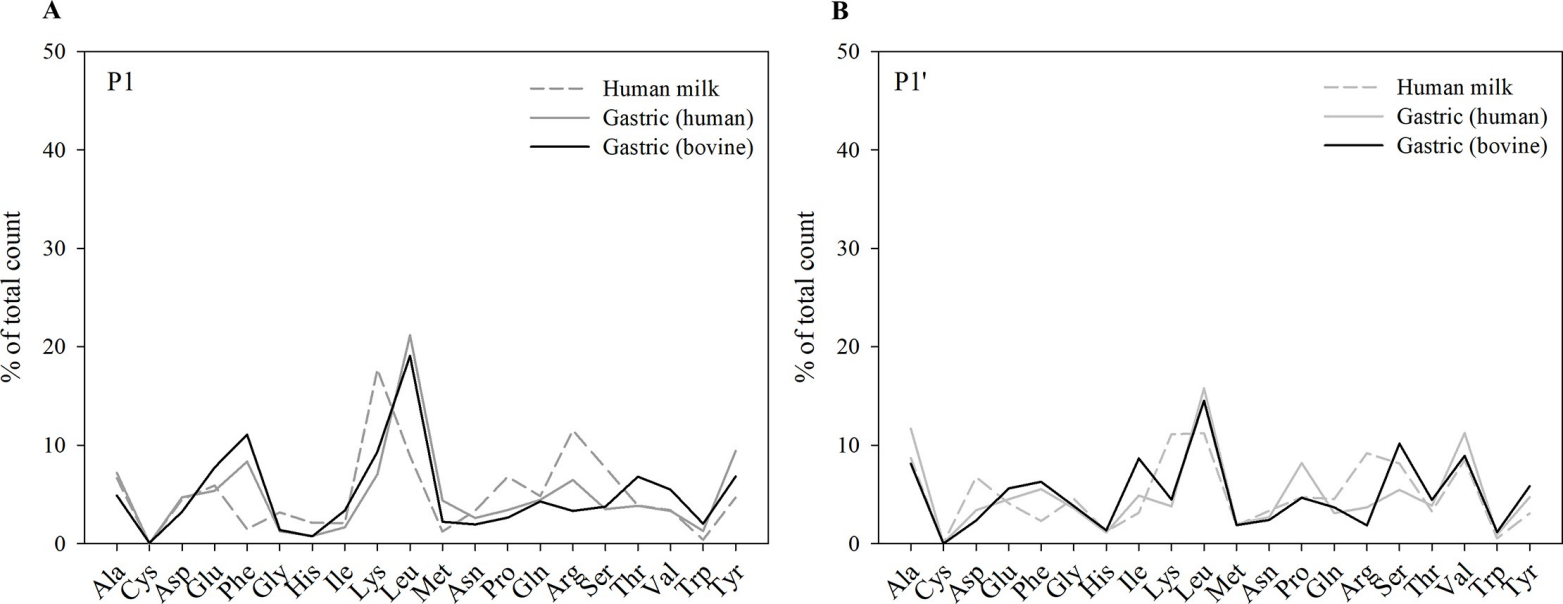
Distribution of amino acids at P1 and P1’ position by count. Mean relative count of (A) P1 and (B) P1’ positions of human and bovine milk protein-derived peptides identified in five human milk and infant gastric samples distributed by amino acid.

**Fig 5:**
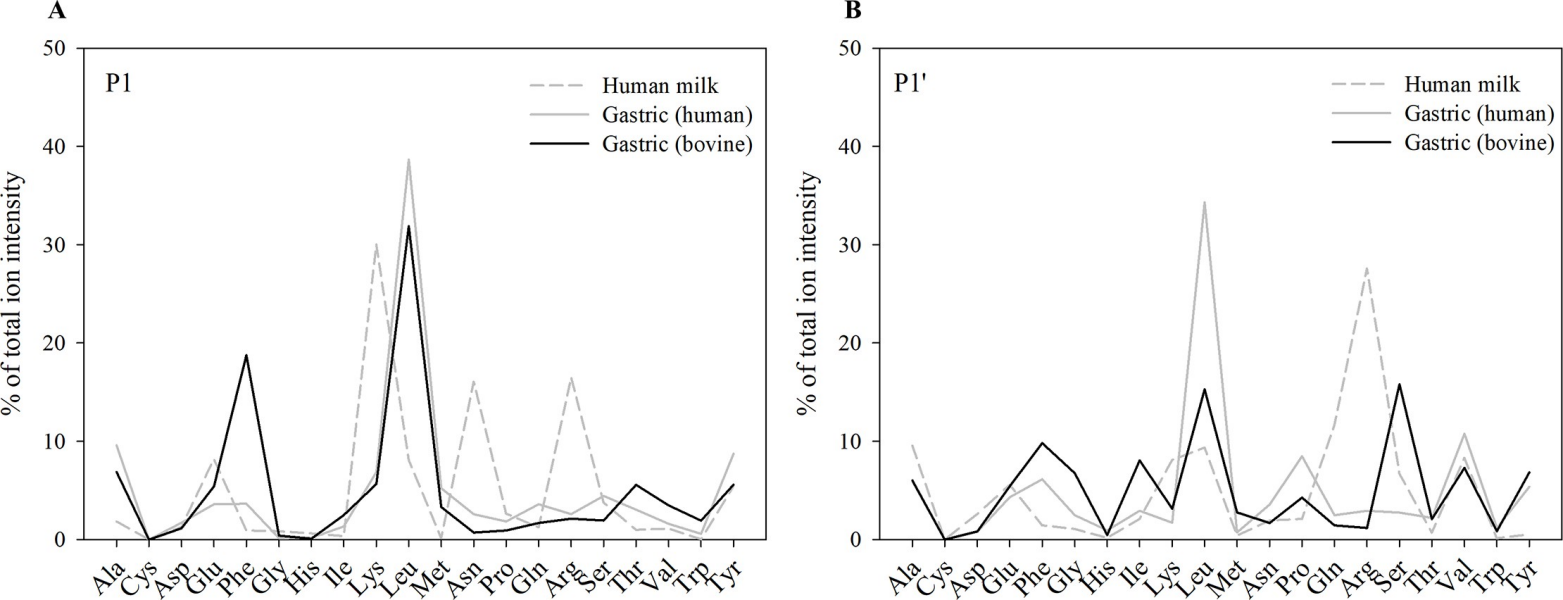
Distribution of amino acids at P1 and P1’ position by ion intensity. Mean relative ion intensity of (A) P1 and (B) P1’ positions of human and bovine milk protein-derived peptides identified in human milk and infant gastric samples distributed by amino acid.

Each of the 692 ± 7 and 981 ± 101 peptides identified from bovine and human milk proteins in the gastric samples of infants, respectively, have both an N- and C-terminus, resulting in potentially double the number of cleavage sites as peptides identified. However, of these peptides, 83 ± 6 bovine milk peptides and 82 ± 5 human milk peptides had a C- or N-terminus, which was the same as the parent protein’s C- or N-terminus, and therefore did not count as a cleavage site. The amino acids present before and after a cleavage site were matched to the cleavage site specificity of proteases to determine which proteases most likely cleaved each peptide bond. For most proteases, the amino acids at P1 and P1’ are of most importance for peptide cleavage specificity. The most common P1 position amino acids in human milk (by relative count and relative ion intensity, respectively) were Lys (17.7%, 30.0%), Arg (11.5%, 16.5%), Leu (8.9%, 0.1%) and Ser (7.7%, 3.8%). The high level of P1 Lys and Arg observed matches the sequence specificity for plasmin. The remaining cleavage site P1 positions were distributed among all other amino acids except for Cys, which was never present at the P1 position of a cleavage site (Fig 4 and Fig 5).

In the infant gastric samples, for peptides deriving from human milk proteins, the most common P1 site amino acids were Leu (21.2%, 38.7%), Tyr (9.4%, 8.7%)), Phe (8.3%, 3.7%) and Ala (7.2%, 9.6%). For the bovine peptides in the infant gastric samples, the most common P1 site amino acids were Leu (19.1%, 31.9%), Phe (11.1%, 18.8%), Glu (7.7%, 5.4%), Tyr (6.8%, 5.6%) and Trp (6.8%, 5.6%) (Fig 4 and Fig 5). Cysteine was never present at the P1 or P1’ position of a cleavage site. Phe and Leu are preferred amino acids at P1 for pepsin and cathepsin D.

Proteasix is an online software that predicts the protease responsible for peptide cleavage based on the P4–P4’ amino acids positioned around the cleavage site [40]. In the Proteasix analysis, most peptides identified in the milk and gastric samples were not assigned to a specific protease. Of those matched, Proteasix predicted most of the cleavage sites of human milk proteins to derive from the activity of plasmin (49 ± 3 cleavage sites) and elastase (23 ± 4 cleavage sites). Additional cleavage sites matched to thrombin (10 ± 1 cleavage sites) and kallikrein 6 (3 ± 0 cleavage sites). The relative count of cleavage sites matched to each protease is shown in Table 2.

**Table 2:** Relative amount of cleavage sites assigned to enzymes identified by Proteasix.

	Cathepsin D	Elastase	Thrombin	Kallikrein 6	Pepsin	Plasmin	Cathepsin D/Pepsin	Unassigned
Bovine Gastric	10.0±0.4^a^	5.1±0.3	0.3±0.0	0.1±0.0	6.9±0.3	0.5±0.1	5.0±0.2	72.0±0.4
Human Milk	N/A	3.3±0.2	1.5±0.2	0.5±0.1	N/A	7.2±0.3	N/A	87.5±0.4
Human Gastric	5.5±0.4	4.7±0.4	0.5±0.1	0.3±0.2	4.8±0.7	2.3±0.6	7.4±1.2	74.6±1.0

^a^ Mean ± standard error, *n* = 5

Proteasix predicted that most of the cleavage sites of milk proteins from gastric aspirates derived from the activity of cathepsin D (93 ± 5 in bovine milk proteins, 75 ± 12 in human milk proteins) and pepsin (64 ± 3, 67 ± 14). Many of the cleavage sites could be assigned to either cathepsin D or pepsin, as the cleavage site specificities of these two enzymes overlap and cannot always be distinguished by this method. On average, 47 ± 2 and 105 ± 25 of bovine and human milk protein cleavage sites, respectively, could be assigned to both cathepsin D and pepsin. Some gastric peptide cleavage sites matched to elastase (48 ± 2 in bovine proteins, 60 ± 2 in human), and a low number matched to plasmin (5 ± 1, 27 ± 4), thrombin (3 ± 0, 6 ± 1) and kallikrein 6 (1 ± 0, 3 ± 1).

### Bioactive peptides

Searching the identified peptides against the MBPDB revealed 58 peptides that were identical with known bioactive peptides: 5 bioactive peptides deriving from human milk proteins, 50 from bovine milk proteins, and 3 that could not be distinguished between the two species due to identical sequences (Table 3). All five of the human milk peptides were identified in the gastric samples, but only two were identified in the milk samples. No bioactive human milk protein-derived peptides were present in either all five of the milk samples or all five of the gastric samples. Eleven of the 100% homologous bovine peptides were identified in all five gastric samples and are listed in bold in Table 3.

**Table 3:** Peptides in the milk or gastric samples with 100% homology to known bioactive peptides.

Species	Query peptide	Protein	Milk	HMF	Gastric	Function
Both	ENLHLPLP	β-casein			X	ACE-inhibitory
	ENLHLPLPLL	β-casein			X	ACE-inhibitory
	LHLPLPL	β-casein			X	ACE-inhibitory
Bovine	AEIYGTKESPQTHYY	Lactoferrin			X	Stimulates Proliferation
	AYFYPEL	αs1-casein			X	ACE-inhibitory, Antioxidant, Increase MUC5AC Expression, Opioid
	DAYPSGAW	αs1-casein			X	ACE-inhibitory
	FPEVFGK	αs1-casein		X		ACE-inhibitory
	FVAPFPEVFG	αs1-casein			X	ACE-inhibitory
	HIQKEDVPSERYLGYLEQLLRLK	αs1-casein		X		Antimicrobial
	LAYFYPEL	αs1-casein			X	Immunomodulatory
	**LRLKKYKVPQL**^a^	αs1-casein			X	Antimicrobial
	**RPKHPIKHQGLPQEVLNENLLRF**	αs1-casein			X	Antimicrobial
	SDIPNPIGSENSEK	αs1-casein		X	X	Antimicrobial
	VLNENLLR	αs1-casein			X	Antimicrobial
	KTVYQHQKAMKPWIQPKTKVIPYVRYL	αs2-casein			X	Antimicrobial
	LKKISQRYQKFALPQY	αs2-casein			X	Antimicrobial
	LKTVYQHQKAMKPWIQPKTKVIPYVRYL	αs2-casein			X	Antimicrobial
	PYVRYL	αs2-casein		X	X	ACE-inhibitory, Antimicrobial, Antioxidant
	TKKTKLTEEEKNRL	αs2-casein		X		Antimicrobial
	**TKVIPYVRYL**	αs2-casein		X	X	Antimicrobial
	VYQHQKAMKPWIQPKTKVIPYVRYL	αs2-casein			X	Antimicrobial
	**YQKFPQY**	αs2-casein			X	ACE-inhibitory, Antioxidant
	EPVLGPVRGPFP	β-casein		X	X	ACE-inhibitory
	**LLYQEPVLGPVRGPFPIIV**	β-casein		X	X	ACE-inhibitory
	LNVPGEIVE	β-casein			X	ACE-inhibitory
	LVYPFPGPIP	β-casein			X	ACE-inhibitory
	NIPPLTQTPV	β-casein		X		ACE-inhibitory
	**PVVVPPFLQPE**	β-casein			X	Antimicrobial
	QEPVLGPVRGPFPIIV	β-casein			X	ACE-inhibitory
	RDMPIQAF	β-casein		X		ACE-inhibitory
	RELEELNVPGEIVESLSSSEESITR	β-casein		X	X	Caseinophosphopeptide
	VLPVPQKAVPYPQR	β-casein			X	Antimicrobial
	VYPFPGPIP	β-casein			X	PEP-inhibitory
	VYPFPGPIPN	β-casein			X	ACE-inhibitory, Antioxidant
	YQEPVLGPVR	β-casein		X	X	ACE-inhibitory
	YQEPVLGPVRG	β-casein			X	ACE-inhibitory
	**YQEPVLGPVRGPFPI**	β-casein			X	Antimicrobial
	**YQEPVLGPVRGPFPIIV**	β-casein		X	X	ACE-inhibitory, Antimicrobial, Antithrombin, Immunomodulatory
	AASDISLLDAQSAPLR	β-lactoglobulin			X	Antimicrobial
	DAQSAPLRVY	β-lactoglobulin			X	ACE-inhibitory
	GLDIQKVAGT	β-lactoglobulin			X	Antimicrobial
	IIAEKTKIPAVF	β-lactoglobulin			X	Antimicrobial
	IPAVFKIDA	β-lactoglobulin			X	DPP-IV-Inhibitory
	IQKVAGTW	β-lactoglobulin			X	ACE-inhibitory, DPP-IV-Inhibitory
	**LDIQKVAGTW**	β-lactoglobulin			X	ACE-inhibitory
	LIVTQTMK	β-lactoglobulin			X	Cytotoxic
	LKPTPEGDLE	β-lactoglobulin			X	DPP-IV-Inhibitory
	SLAMAASDISLL	β-lactoglobulin			X	Antimicrobial
	TPEVDDEALEK	β-lactoglobulin		X		Antimicrobial, DPP-IV-Inhibitory
	HPHPHLSF	κ-casein			X	ACE-inhibitory
	INNQFLPYPY	κ-casein			X	DPP-IV-Inhibitory
	**MAIPPKKNQDKTEIPTINT**	κ-casein			X	Antimicrobial
	**VQVTSTAV**	κ-casein			X	Antimicrobial
Human	LENLHLPLP	β-casein			X	ACE-inhibitory
	QELLLNPTHQIYPVTQPLAPVHNPISV	β-casein	X		X	Antimicrobial
	SPTIPFFDPQIPK	β-casein	X		X	Stimulates Proliferation
	WSVPQPK	β-casein			X	ACE-inhibitory, Antioxidant
	YANPAVVRP	κ-casein			X	ACE-inhibitory

^a^ Bolded peptides were present in all five samples.

In addition to the identical sequences, multiple peptides identified in the milk and gastric samples were highly homologous (≥ 80% sequence match) to known bioactive peptides. Several of these peptides were identified with more than one function. The high degree of sequence similarity suggests the possibility for these peptides to have bioactive function as well. We identified 85 bovine milk peptides in the HMF with ≥ 80% homology to a known bioactive peptide. In the human milk, 31.2 ± 3.3 peptides with ≥ 80% homology to a known bioactive peptide were identified as deriving from human milk proteins. Of those, 11 peptides were identified in all 5 milk samples. In the gastric samples, a total of 206.4 ± 8.6 peptides with ≥ 80% homology to a known bioactive peptide were identified. From the total, 165.6 ± 5.9 were identified from bovine proteins, and 40.8 ± 3.8 were identified from human proteins. Fifty bovine peptides and eleven human peptides were present in all five gastric samples.

**Fig 6:**
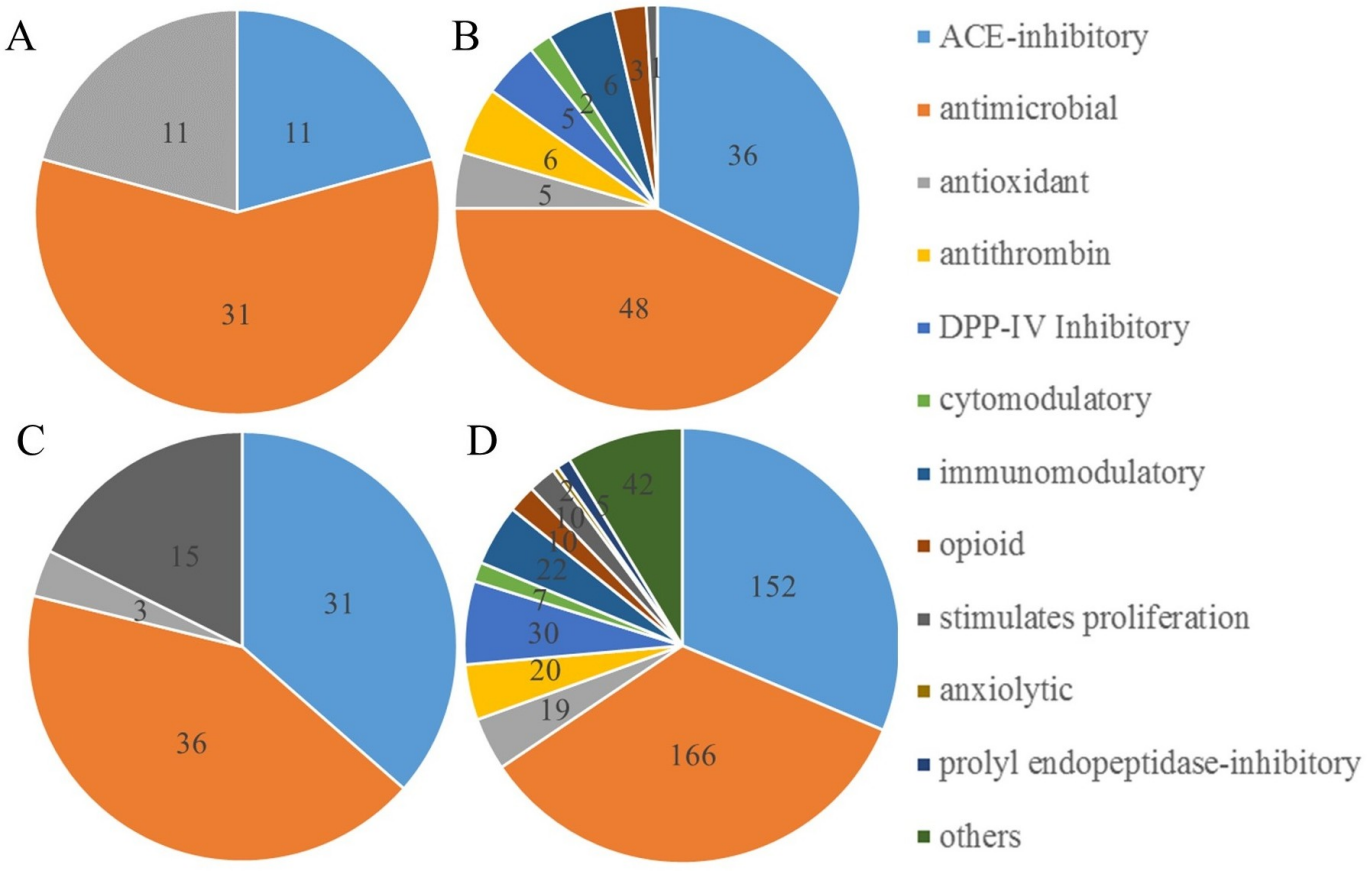
Bioactive peptides identified in human milk or infant gastric. Bioactive peptides identified in the (A) milk, (B) bovine-based HMF, (C) gastric samples (human-derived) and (D) gastric samples (bovine-derived) by searching the Milk Bioactive Peptide Database (MBPDB), with a threshold value of ≥ 80% homology.

The counts and total ion intensity of human-derived bioactive peptides significantly increased from human milk to the gastric samples (*P* = 0.0438 and *P* = 0.00287, respectively). The count and total ion intensity of bovine-derived bioactive peptides increased from HMF to gastric samples, 212% and 2268%, respectively; however, as only one sample of HMF was analyzed, no statistical power could be calculated. Peptides highly similar to known antimicrobial peptides were the most often identified, including 31 from human milk, 48 from HMF, 36 from human milk proteins in the gastric samples and 166 from bovine milk proteins in the gastric samples (Fig 6). Peptides with sequences closely related to known ACE-inhibitory peptides were often identified: 11, 36, 31 and 152 possible ACE-inhibitory peptides were identified in human milk, HMF, gastric (human) and gastric (bovine) samples, respectively. Additional peptides with potential antioxidant and cell-proliferation stimulatory effects were identified from human milk proteins. From bovine milk proteins, bioactive peptides with a greater variety of functions were identified, including DPP-IV-inhibitory, antithrombotic, cytomodulatory, immunomodulatory, opioid and anxiolytic peptides (S1 Table).

## Discussion

This study profiled the peptides in human milk, a bovine milk-based fortifier and the gastric contents of preterm infants after fortified human milk feedings. Peptides from both human milk proteins and bovine fortifier proteins increased from the undigested feed to the stomach. The total count of human milk protein-derived peptides increased from the milk to gastric samples, and the total count of bovine milk protein-derived peptides increased from the HMF to gastric samples. Total peptide ion intensity also increased from milk/HMF to the gastric samples for both protein types. A previous study in term infants also showed an increase in peptide counts and ion intensity from milk to gastric samples [14]. Use of the Orbitrap Fusion Lumos mass spectrometer allowed us to identify far more peptides than were previously discovered (5,264 compared to 418 [14]). The current study used database searching for the identification of peptides. This approach will not identify peptides shorter than seven amino acids, but it is likely that gastric digestion results in several short peptides that were not detected.

Peptides that were identified from all five mother-infant pairs accounted for the majority of the peptide ion intensity for both milk and gastric samples. The finding that the majority of the human milk peptide ion intensity derived from peptides found in all five milks matches previous findings from foremilk and hindmilk [34]. In the gastric samples, the majority of both human and bovine protein-derived peptide ion intensity was conserved in all five infants. However, the majority of peptide count from both species was composed of peptides that were found in fewer than all five of the samples, with human milk protein-derived peptides being slightly less conserved. The differences in count likely resulted from small variations in protease activity that differentially released peptides from the parent proteins. Though the abundance of these peptides was not large, there is the possibility that the sequence differences had varying bioactivity.

Caseins represent about 20% of milk proteins in early lactation and 45% of milk proteins in late lactation [41]. Of the human milk peptides, β-casein accounted for 71.2% of the total human peptide ion intensity, with α_s1_-casein and κ-casein combining for an additional 11.7%. Likewise, HMF has a blend of milk protein and whey proteins designed to match the casein:whey ratio of human milk [42], yet the HMF-derived bovine peptides from caseins accounted for 84.9% of the gastric peptide ion intensity. Using peptide ion intensity as an indicator for relative gastric protein digestion, the casein proteins in both HMF and human milk, and β-casein in particular, were digested more efficiently than the whey proteins. These findings match, in part, with findings of the previous study on term infant gastric digestion [14], except that in that study, lactoferrin-derived peptides made up a relatively larger proportion of the peptides released in the stomach. That difference could indicate that the term infant stomach has greater potential to degrade whey proteins than the preterm infant stomach. A caveat is that using peptide ion intensity can only be used as an estimate of true abundance, as peptide ionization efficiency varies between peptides.

The combination of human milk and bovine-based fortifier in the sampled preterm infants’ diets allowed for comparison of gastric digestion between human- and bovine-derived proteins. The protein profile closely followed that found in previous human and bovine milk studies, and in vitro gastric digestion studies [37, 43, 44]. β-casein from both species was the most digested protein based on peptides released, while α_s1_-casein-derived peptides from bovine milk were relatively more abundant than the human counterpart. Though osteopontin was partially digested in human milk and was the third most hydrolyzed protein in the gastric samples, by count, few peptides from bovine osteopontin were identified in the gastric samples. The cleavage pattern between β-casein, α_s1_-casein and osteopontin was highly dissimilar between human and bovine proteins, as shown by alignment of the peptide distribution.

Previously, we measured the concentration and activity of proteases in both human milk and gastric aspirates of infants [9]. Our findings in this study contribute to determining the end-products of protease activity in the preterm infant stomach. From the P1–P1’ amino acid annotations in human milk, we found that Lys and Arg were mostly observed at the P1 position of a cleavage site. Plasmin, kallikrein and thrombin are enzymes known to be active in human milk [9, 16] that prefer to cleave after these amino acids. Hence, it is not easy to distinguish between these enzymes based on the P1 amino acid. The bioinformatics tool Proteasix uses a more complex algorithm to identify enzyme activity and takes into account amino acids from P4 to P4’. This analysis found that most cleavage sites in the milk samples matched to the specificity of plasmin (above that of kallikrein 6 and thrombin). A recent peptidomics study of preterm and term milk samples also indicated that, based on cleavage sites, plasmin was highly active within milk [16].

In the gastric samples, the P1–P1’ amino acid annotations switched from mostly Lys and Arg in human milk to Leu and Phe in the stomach, which matches the P1 amino acid cleavage specificity of pepsin/cathepsin D. The Proteasix P4 to P4’ analysis showed that both cathepsin D and pepsin are likely highly active in cleaving both bovine and human milk proteins in the preterm infant stomach. As cathepsin D and pepsin both preferentially cleave after a Leu/Phe amino acid, a large portion of the predicted cleavages sites could be assigned to either cathepsin D or pepsin. We previously observed that milk cathepsin D is inactive within human milk but becomes active when exposed to the more acidic pH of the infant stomach [16]. This activation is expected as acidic conditions lead to autoactivation of the inactive procathepsin D to the active pseudocathepsin D [45]. Pepsin is known to be produced in the infant stomach as early as 16 weeks of gestation [46]. These data demonstrate a clear shift in the release of milk peptides from the activity of mostly plasmin in human milk to mostly pepsin and cathepsin D in the stomach. The addition of fortifier is unlikely to contribute to the protease activity, as these enzymes would be most likely inactivated by the heat-treatment during processing [47].

A large number of cleavage sites could not be assigned to the proteases examined. These cleavage sites could potentially be due to the activity of cytosol aminopeptidase and carboxypeptidase B2, which are exopeptidases that sequentially release amino acids from the N- and C-termini, respectively. These enzymes are known to be present in milk [9], and based on peptide profiling, are thought to be major contributors to milk protein digestion [48]. Human milk protein-derived peptides and bovine milk protein-derived peptides had similar numbers of cleavage sites that could be due to exopeptidase activity.

This study demonstrated that a large number of potentially bioactive peptides are released by milk proteases in the mammary gland and the preterm infant stomach. Potential bioactive peptides increased from the undigested milk/fortifier to the stomach for both human and bovine proteins. Whether these peptides exert their bioactive effects depends on whether they reach their site of action. Further proteolytic activity in the intestine due to the action of pancreatic proteases and brush-border peptidases is likely to break down existing peptides and release new ones from the milk proteins [49]. Studies investigating the systemic effect of milk-derived bioactive peptides are limited, but several of the identified predicted bioactive peptides may be relevant for the gut, such as those with antimicrobial and mucin-stimulatory properties.

A higher number of potential bioactive peptides from bovine milk proteins were identified compared with those from human milk proteins. The predominance of bovine milk-derived bioactive peptides most likely derives from the fact that bovine milk has been more comprehensively studied for bioactivity than has human milk rather than any biological reality. The MBPDB contains 713 known bovine milk bioactive peptides and only 106 known human milk bioactive peptides [33]. However, though more than twice as many ACE-inhibitory peptides than antimicrobial peptides are described in the literature (and present in the database) [33], more peptides homologous to known antimicrobial peptides were identified in the samples in the present study, which demonstrates that determining homology does not reflect a simple probability distribution. Several literature-described bioactive peptides have a short sequence of fewer than seven amino acids, which is below the size threshold for the peptide analysis approach used in this study and therefore are not identified.

In conclusion, proteins from human milk and bovine milk proteins from HMF were similarly digested in the preterm infant stomach. Both human milk and HMF contain bioactive peptides, and the number, abundance and functional variety of both human and bovine-derived peptides increased with gastric digestion in preterm infants. Studies of the potential clinical impact of bioactive peptides released in the premature infant stomach will be of particular importance, including impact on the many diseases of prematurity in which inflammation and/or oxidants play a role such as peri-ventricular leukomalacia, bronchopulmonary dysplasia, retinopathy of prematurity, and necrotizing enterocolitis. Also of interest will be the impact of released proteins on more typical nutritional outcomes such as growth, neurodevelopment, anemia of prematurity and metabolic bone disease of prematurity. The addition of analysis of gastric aspirates for bioactive peptides to current cohort studies focused on the diseases unique to premature infants has great potential value.

## Supporting information

S1 Table The complete list of peptides identified in the HMF, milk, and gastric samples. Sequence lists the peptide sequence. Species lists the peptide's parent protein species. Protein Name lists the parent protein. Start and Stop list the amino acid position the peptide is derived from in the parent protein. HMF, Milk, and Gastric list the number of samples the peptide was identified in. Modifications lists all modifications the peptide was identified with (O = Oxidation, P = Phosphorylation, A = Acetylation). Highest Homology Match lists the bioactive peptide from the MBPDB that the peptide most closely matched by sequence. % Alignment lists the sequence alignment. Function lists the bioactive functions of homologous peptides. DOI lists the journal article of the Highest Homology Match.
(XLSX)
